# Supplementary material for: PRState: Incorporating genetic ancestry in prostate cancer risk scores for men of African ancestry
Source: BMC Cancer. 2022 Dec 9;22:1289. doi: 10.1186/s12885-022-10258-3 (PMC9733391; doi:10.1186/s12885-022-10258-3)
Supplement: Supplementary file 2 — Additional file 2. Supplemental Figure 1: Performance of Polygenic Risk Scores Constructed from Different Ancestral Backgrounds in African Ancestry Group in ELLIPSE Consortium. ROC curve for genetic prediction of prostate cancer risk in ELLIPSE Consortium African ancestry group (n=4,533) using: (A) Polygenic risk scores constructed from 10 African ancestry-specific variants only. (B) Polygenic risk scores constructed from 7 European ancestry-specific variants only. (C) Polygenic risk scores constructed from 14 trans-ancestry specific variants only. Figure 2: Comparison of African Prostate Cancer Variant Odds Ratios in European, African, and Trans-Ancestry Groups in ELLIPSE Consortium. Odds ratios for 10 African ancestry-specific PrCa variants for prostate cancer in the ELLIPSE Consortium for European (n=5,667), African (n=4,553) and Trans-Ancestry (n=10,100) ancestry groups. Figure 3: Comparison of African Prostate Cancer Variant Odds Ratios in European and African HAREGroups in Million Veteran Program. Odds ratios for 10 African ancestry-specific PrCa variants for any prostate cancer (A), fatal prostate cancer (B), and metastatic prostate cancer (C) in the African (n=121,964) and European (n=461,627) HARE Million Veteran Program groups. Figure 4: Evaluation of PRState Score in Million Veteran Program Including Socioeconomic Information. (A) Odds ratio of PRState with prostate cancer clinical endpoints when socioeconomic variables were included (green) versus not (orange). Socioeconomic variable include income (>$50,000) and education level (at least bachelor’s degree). ROC-AUCanalysis for any (B), metastatic (C) and fatal (D) prostate cancer divided by socioeconomic group. High socioeconomic group are African ancestry individuals with income >$50,000 and at least bachelor’s degree. Low socioeconomic group are African ancestry individuals with income <$50,000 and education level less than bachelor’s degree. Figure 5: Evaluation of PRState Score in Million Veteran Pr [file 12885_2022_10258_MOESM2_ESM.docx]

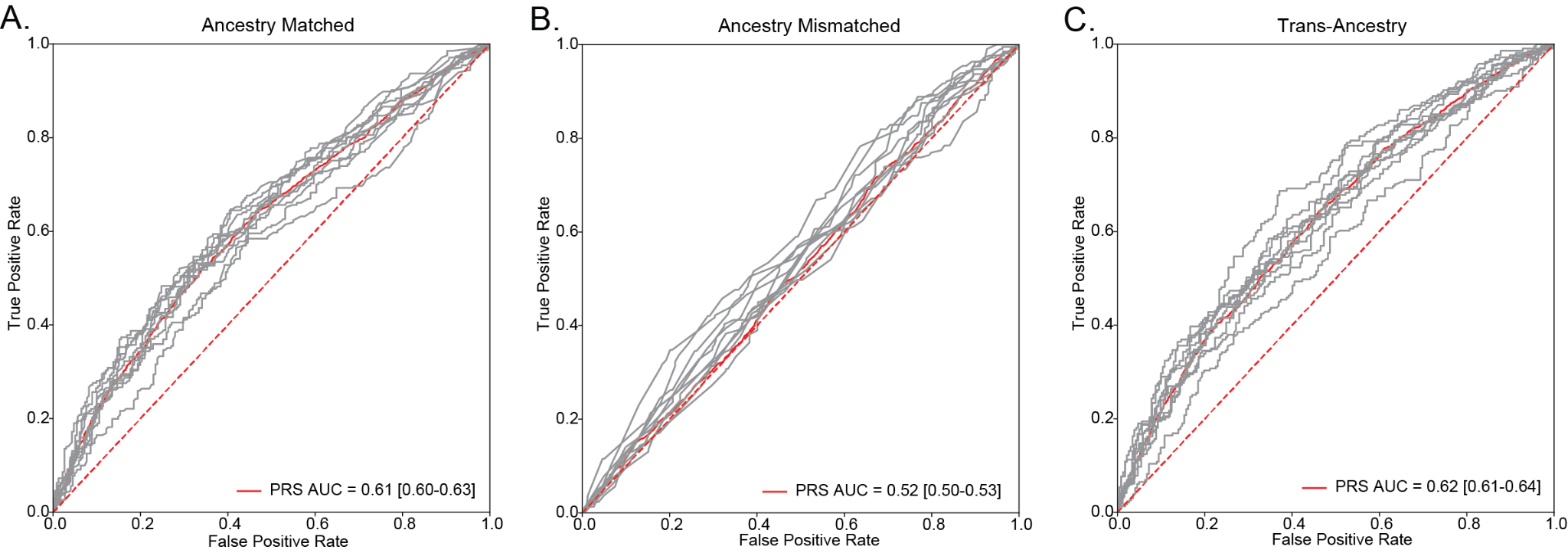


**Supplemental Figure 1: Performance of Polygenic Risk Scores Constructed from Different Ancestral Backgrounds in African Ancestry Group in ELLIPSE Consortium.** ROC curve for genetic prediction of prostate cancer risk in ELLIPSE Consortium African ancestry group (n=4,533) using: **(A)** Polygenic risk scores constructed from 10 African ancestry-specific variants only. **(B)** Polygenic risk scores constructed from 7 European ancestry-specific variants only. **(C)** Polygenic risk scores constructed from 14 trans-ancestry specific variants only.

**
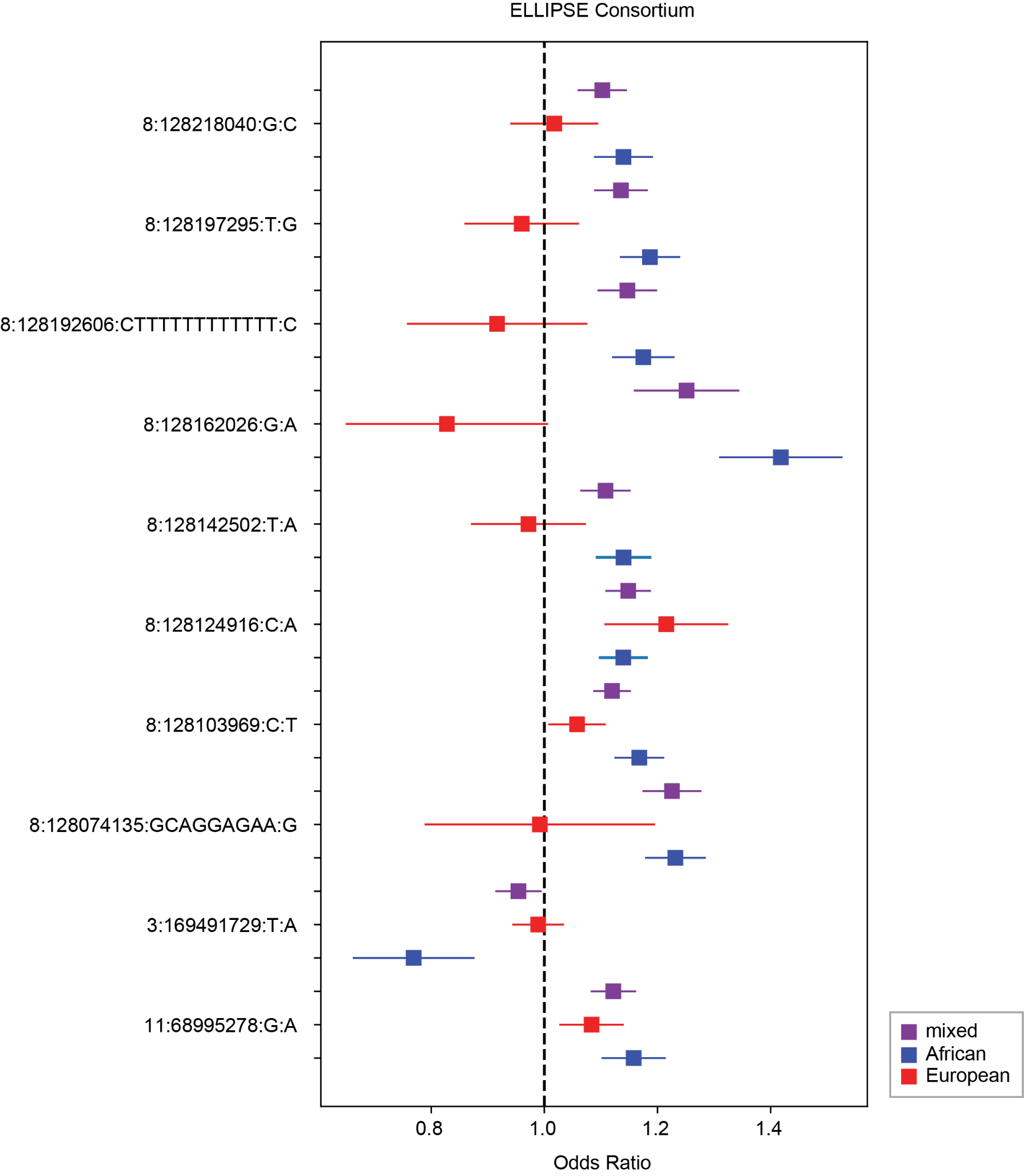
**

**Supplemental Figure 2: Comparison of African Prostate Cancer Variant Odds Ratios in European, African, and Trans-Ancestry Groups in ELLIPSE Consortium.** Odds ratios for 10 African ancestry-specific PrCa variants for prostate cancer in the ELLIPSE Consortium for European (n=5,667), African (n=4,553) and Trans-Ancestry (n=10,100) ancestry groups.

**
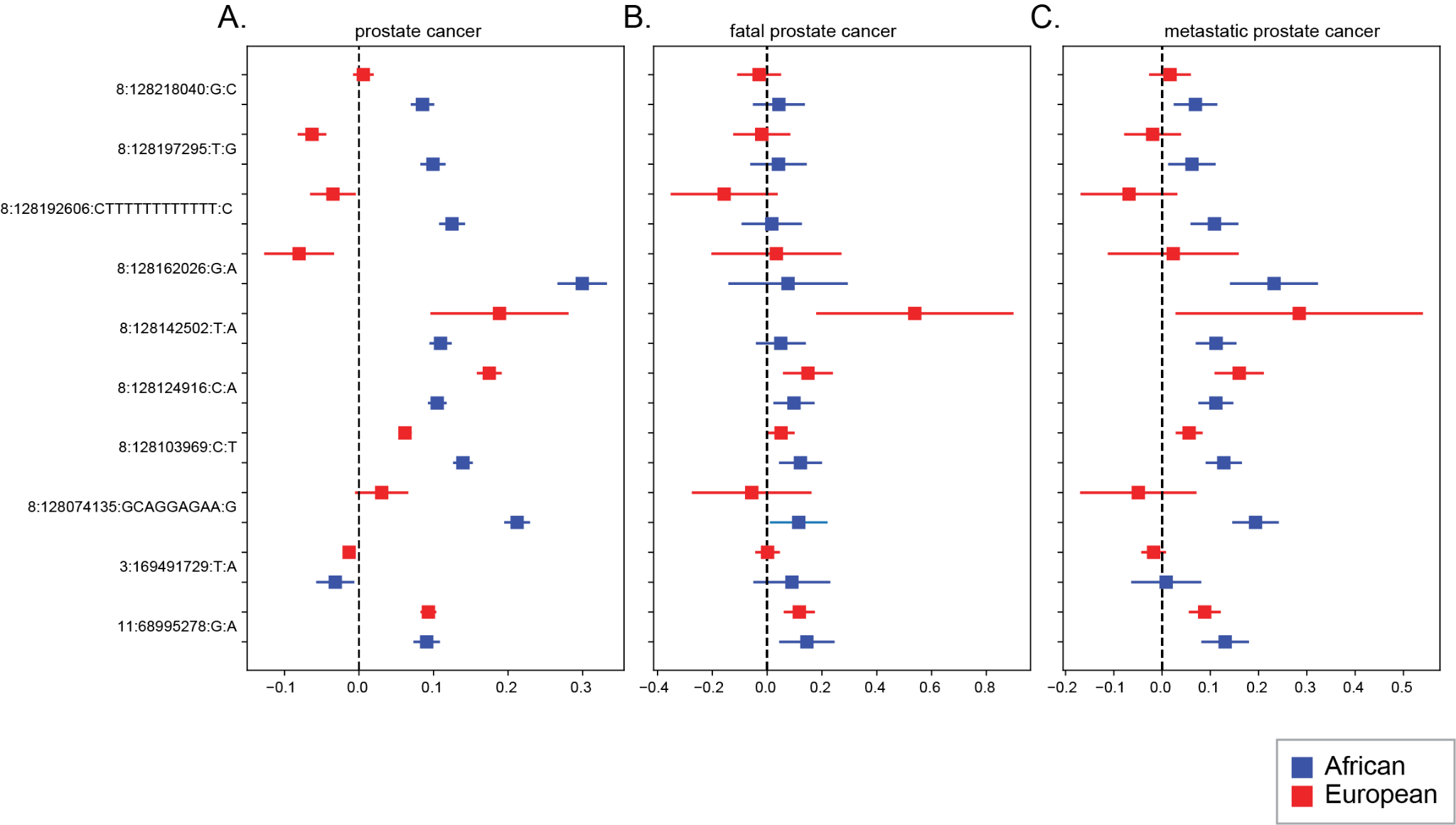
**

**Supplemental Figure 3: Comparison of African Prostate Cancer Variant Odds Ratios in European and African HARE Groups in Million Veteran Program.** Odds ratios for 10 African ancestry-specific PrCa variants for any prostate cancer **(A),** fatal prostate cancer **(B),** and metastatic prostate cancer **(C)** in the African (n=121,964) and European (n=461,627) HARE Million Veteran Program groups.


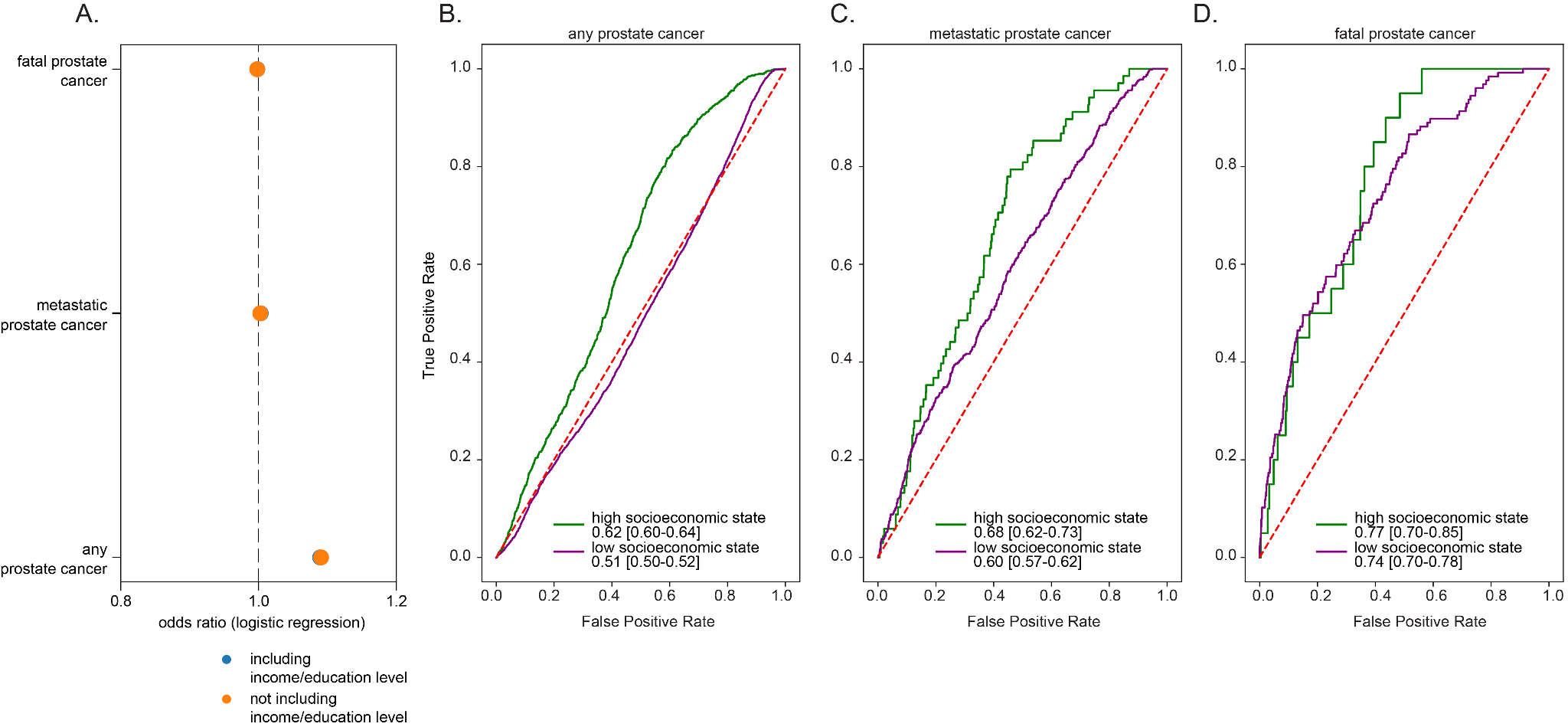


**Supplemental Figure 4: Evaluation of PRState Score in Million Veteran Program Including Socioeconomic Information. (A)** Odds ratio of PRState with prostate cancer clinical endpoints when socioeconomic variables were included (green) versus not (orange). Socioeconomic variable include income (>$50,000) and education level (at least bachelor’s degree). ROC-AUC analysis for any **(B),** metastatic **(C)** and fatal **(D)** prostate cancer divided by socioeconomic group. High socioeconomic group are African ancestry individuals with income >$50,000 and at least bachelor’s degree. Low socioeconomic group are African ancestry individuals with income <$50,000 and education level less than bachelor’s degree

**
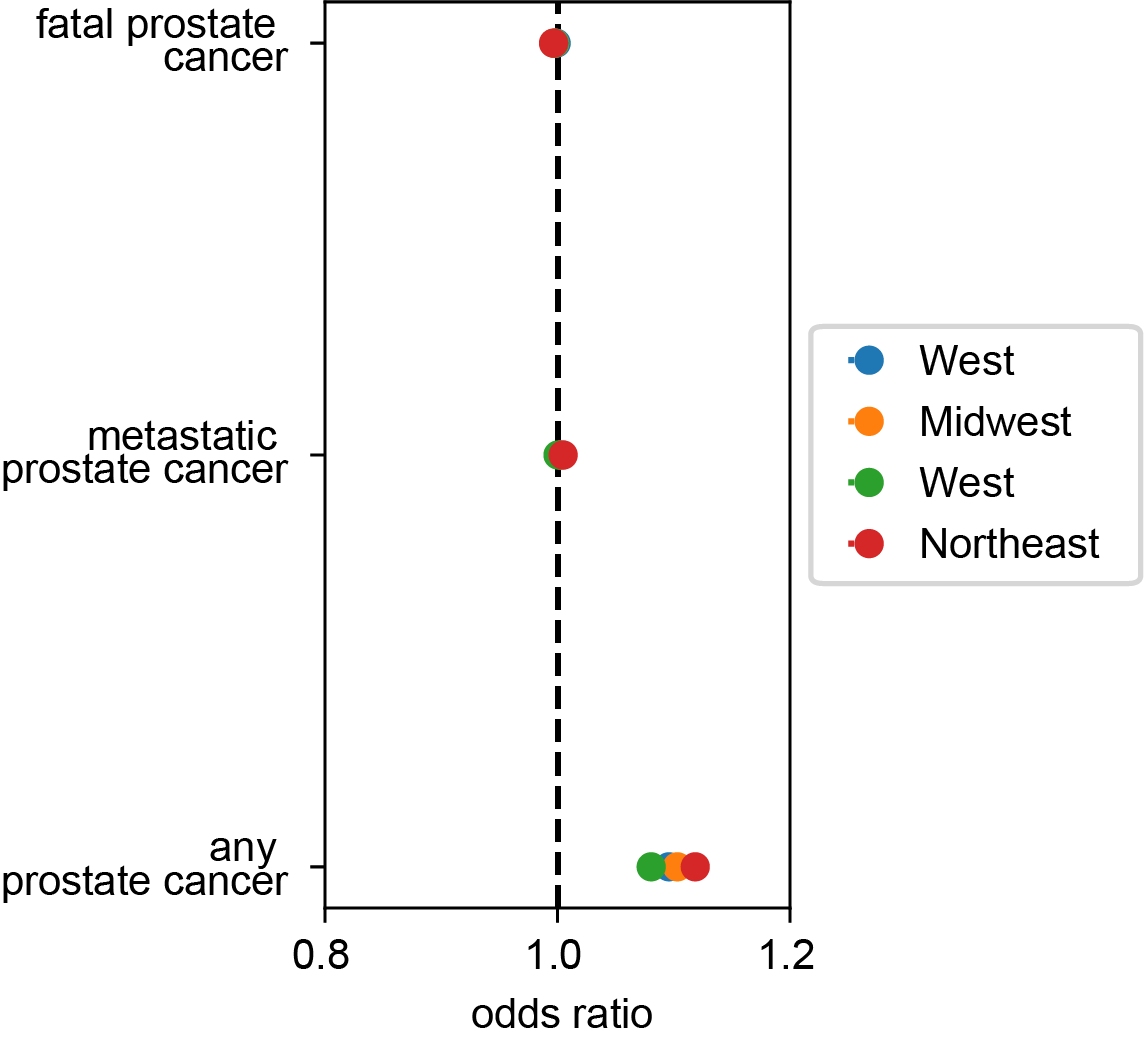
**

**Supplemental Figure 5: Evaluation of PRState Score in Million Veteran Program Based On Geographic Region.** Odds ratio of PRState with prostate cancer clinical endpoints divided by VA enrollment site geographical region.
